# Health-related quality of life among thalassemia patients in Bangladesh using the SF-36 questionnaire

**DOI:** 10.1038/s41598-023-34205-9

**Published:** 2023-05-12

**Authors:** Md Jubayer Hossain, Md Wahidul Islam, Ummi Rukaiya Munni, Rubaiya Gulshan, Sumaiya Akter Mukta, Md Sharif Miah, Sabia Sultana, Mousumi Karmakar, Jannatul Ferdous, Mohammad Ariful Islam

**Affiliations:** 1 Population Health Studies Division, Center for Health Innovation, Research, Action, and Learning-Bangladesh (CHIRAL Bangladesh), 9-10 Chittaranjan Avenue, Dhaka, 1100 Bangladesh; 2 Health Systems and Population Studies Division, International Centre for Diarrhoeal Disease Research, Bangladesh, 68, Shaheed Tajuddin Ahmed Sarani Mohakhali, Dhaka, 1212 Bangladesh; 3 BRAC James P Grant School of Public Health, BRAC University, 66, Mohakhali, Dhaka, 1212 Bangladesh; 4 Department of Microbiology, Jagannath University, 9-10 Chittaranjan Avenue, Dhaka, 1100 Bangladesh; 5 Department of Microbiology, Bangladesh University of Health Sciences, 125, Technical Mor, 1 Darus Salam Rd, Dhaka, 1216 Bangladesh; 6 Department of Transfusion Medicine, Mugda Medical College and Hospital, Hazi Kadam Ali Rd, Dhaka, Bangladesh; 7 Bangladesh Thalassemia Foundation, Chamelibagh, Shantinagar, Dhaka, 1217 Bangladesh

**Keywords:** Health policy, Health services, Public health

## Abstract

Thalassemia is one of the most common autosomal recessive hereditary blood disorders worldwide, especially in developing countries, including Bangladesh. Thus, this study aimed to determine HRQoL and its determinants of thalassemia patients (TP) in Bangladesh. A cross-sectional survey was performed on 356 randomly selected thalassemia patients. Participants were invited to face-to-face interviews. Descriptive statistics (frequencies and percentages), independent t-test, ANOVA, and multivariate (linear and logistic regression) analysis was performed to analyze the data. Our demographic data showed that among 356 patients, 54% and 46% were male and female, respectively, with an average age of 19.75 (SD = 8.02) years. Most were transfusion-dependent (91%), 26% had comorbidities, and 52% were from low-income families. In the case of HRQoL, male patients showed significantly higher scores of bodily pains and physical health summaries than female patients. Lower income, high blood transfusion status, disease severity, comorbidities, and medical expenses (*p* < 0.05; CI 95%) are significantly associated with lower SF-36 scores. This study found an association between lower income, blood transfusion, disease severity, comorbidities, as well as medical expenses, and the deterioration of HRQoL among TP. Male patients experienced poorer HRQoL than females. National action plans are required to guarantee the holistic welfare of thalassemia patients.

## Introduction

Thalassemia is the most common hereditary blood disease, causing anemia worldwide^1,2^. Reduced or absent production of either the α-like or the β-like globin chains to form hemoglobin tetramers during fetal and postnatal life is the main characteristic of this disease^3^. It is identified as the most common chronic genetic disease by the World Health Organization (WHO)^4^ in 60 countries, especially in the Mediterranean region, the Middle East, and Southeast Asia^5–7^. Interestingly, about 300 million people worldwide are carriers of this hemoglobin disorder^6^, and 55 million are in Southeast Asia^8^. For example, in Bangladesh, one member of Southeast Asia, with a population of 160 million population^9^, about 3% are β-thalassemia carriers, and 4% are hemoglobin E (HbE) carriers^10^. The prevalence rate of β-thalassemia, and hemoglobin E (HbE) is 4.1% and 6.1%, respectively^10–12^. Thus, thalassemia becomes a severe public health concern and affects patients negatively.

Previous studies demonstrated that thalassemia causes severe clinical, physical, social, and psychological complications^13^. For instance, poor physical health can result in deformities, growth issues, delayed puberty^14–16^, bone abnormalities, and short height^15,16^. It was also reported that TP has suffered from psychological and social behavior abnormalities such as low self-esteem, depressive symptoms, stress, anxiety, poor social communication, and inadequate school functioning^17^. Furthermore, the TP treatment process, which involves repeated blood draws, multiple transfusions, routine subcutaneous injections, and oral iron chelator therapy, affects their daily life. Consequently, thalassemia reduces their HRQoL^15,18–22^. Therefore, predisposing factors determining the HRQoL of TP might provide a roadmap for mitigating this problem. Evidence-based information on common thalassemia patients' psychological, social, and financial deficits is yet to emerge. The holistic welfare of chronic patients should include varied support initiatives alongside clinical interventions. Potential victims of thalassemia in the young-adult group could be the best target for proactive care support. However, limited studies examined the factors associated with the HRQoL of TP in Bangladesh. We aimed to assess specific indicators of HRQoL among TP using the Short Form-36 (SF-36) questionnaire.

## Methods

### Study design and settings

Thalassemia patients (TP) receiving outpatient treatment at the Bangladesh Thalassemia Foundation were selected for this cross-sectional study (30 Chamelibagh, Shantinagar, Dhaka-1217) from August to November 2021. Study participants with (i) a Bangladeshi resident card, (ii) diagnosed as TP, (iii) aged more than 12 years, and (iv) providing consent were included in this study. On the other hand, study participants under 12 years old and incomplete surveys were excluded from this study. A sample size of 385 participants was estimated.

### Data collection tools

This study assessed HRQoL using the SF-36 (Short Form 36) questionnaire following standard methods^23^. This questionnaire is a well-accepted quality of life (QoL) assessment tool having eight distinct scales and two crucial dimensions. The eight multi-item scales are physical functioning (PF), physical role (RP), bodily pain (BP), general health (GH), vitality (VT), social functioning (SF), emotional role (RE), and mental health (MH). The first five scales are summarized into the physical health dimensions known as the physical health summary (PHS). The last three are summarized into the mental health dimension known as the mental health summary (MHS)^24,25^. Despite being created as a self-administered questionnaire, the SF-36 questionnaire can be completed by interview, telephone, or computer administration^26^. The SF-36 questionnaire was scored quantitatively following the guidelines of the Clinical Outcome Evaluation System software^24,25^. Socio-demographic data such as age, sex, education, marital status, type of family, occupational status, area of residence, and socio-economic status of study participants were collected in this study. Clinical information of participants' TP, including diagnosis, severity, transfusion status, transfusion frequency, previous three-month blood transfusion status, splenectomy status, comorbidities, iron-chelating therapy status, and annual medical expenses, were also collected from medical records, which were collected by clinical staff.

### Data collection procedure

Study participants conducting routine follow-ups at the Bangladesh Thalassemia Foundation were approached. Written informed consent was taken before participating in the study. A clinical record form was prepared for all study participants based on a review of their medical records. The Institutional Review Board (IRB) approval was obtained from the Bangladesh University of Health Sciences study (Memo No: BUHS/ERC/EA/21/37). The Bangladesh Thalassemia Foundation authority also granted the data collection permission. The data collection procedure followed the University of Helsinki ethical guidelines for human subjects research.

### Statistical analysis

Overall, 356 records were included in the final statistical analysis. Descriptive statistics including frequencies and percentages for socio-demographic and clinical information were calculated. The continuous variables were presented as mean ± SD (standard deviations), and categorical variables were presented as proportions. The independent *t*-test was performed to compare means between pairs of patient subgroups relating to sex, age, splenectomy, marital status, comorbidities, and iron chelation. The one-way analysis of variance (One-way ANOVA) was conducted to compare quality-of-life scores in different categories of patients relating to the level of education and economic class. Multivariate analysis using multiple linear regression was used to determine the predictive factors that independently influenced the quality-of-life scores among thalassemia patients. Logistic regression analysis was used to identify factors linked to lower PCS (Physical Component Summary) and MCS (Mental Health Component) scores. A *p*-value threshold of < 0.05 was considered statistically significant at a 95% confidence interval (CI). All statistical analyses were performed using R programming (version 4.2.0). The reproducible table was generated by Gtsummary^27^.

## Results

### Demographic characteristics of the patients

A total of 356 respondents participated in the study, of which 193 (54%) were male, and the remaining 163 (46%) were females. The mean age of the participants was 17 years (ranged 14–24). Most of the patients were students (51%), and their educational status was diverse, but most of them were higher secondary students (59%), followed by primary school students (20.5%) and graduate students (10.9%). Among the respondents, 85% were single, and the majority (51.9%) belonged to the lower-income class. Of the total respondents, 51% of the participants were from urban areas, while the rest participating in the study were from rural communities (villages). The economic classes were divided according to World Bank data^28^. Table 1 demonstrates the demographic characteristics of the patients.

**Table 1:** Demonstrates the demographic characteristics of the patients (N = 365).

Variables	Frequency (%)
Age of participants(years)	19.75 ± 8.02
Gender	
Male	193 (54)
Female	163 (46)
Level of education	
No formal education	7 (2)
Can read and write	27 (7.6)
Primary school	73 (20.5)
Higher Secondary School	210 (59)
Graduate	39 (10.9)
Marital status	
Single	302 (85)
Married	54 (15)
Family types	
Nuclear	211 (59.2)
Joint	142 (40)
Broken	3 (0.8)
Occupational status	
Housewife	17 (4.7)
Student	178 (50.5)
Civil servant/Govt. employee	1 (0.3)
Business	39 (10.9)
Unemployed	121 (33.9)
Area of residence	
Urban	182 (51)
Rural	174 (49)
Socio-economic status	
Low income	185 (51.9)
Moderate income	168 (47.2)
High income	3 (0.8)

### Clinical characteristics of the patients

The clinical characteristic of the 356 thalassemia patients is presented in Table 2. The percentages of patients diagnosed with β-thalassemia major, and others were about 68%, 29.5%, and 1.7% %, respectively. Approximately 54% of the patients presented with severity based on disease complications. About 85% of the patients had received a blood transfusion within three months before the interview, and 91% were transfusion dependent. It was found that the percentage of patients who reported good compliance with iron chelating therapy was 95%.

**Table 2:** Clinical characteristics of thalassemia patients (N = 356).

Characteristics	Frequency (%)
Diagnosis	
E-β thalassemia	242 (68)
β-thalassemia major	105 (29.5)
Haemoglobin H	3 (0.8)
Others	6 (1.7)
Severity (based on disease complication)	
Yes	193 (54)
No	163 (46)
Transfusion status	
Transfusion dependent	323 (90.7)
Transfusion independent	33 (9.3)
Frequency of blood transfusion	
Occasional (1–5 times/year)	64 (18)
Low (6–12 times/year)	156 (44)
High (13–22 times/year)	118 (33)
None	18 (5)
Receiving a blood transfusion during the previous 3 months	
Yes	303 (85)
No	53 (15)
Splenectomy status	
Yes	106 (30)
No	250 (70)
Comorbidities status	
Cardiological disorders	6 (1.7)
Diabetes mellitus	6 (1.7)
Hepatitis B or C	44 (12.3)
No comorbidity	263 (73.87)
Others	37 (10.4)
Iron-chelating therapy status	
Good compliance	337 (95)
Poor compliance	19 (5)
Medical expenses per annum (USD)	559.58 (342, 1139.16)

Others = α thalassemia trait, silent carrier, Hb-Bara.

In addition, it was also found that the lowest and highest yearly medical expenditures were between 342 USD and 1140 USD, respectively. The average annual medical expense was 560 USD.

### Health-related quality of life (QoL) of thalassemia patients

The insights of the SF-36 scores on the eight scales, the two summaries for physical and mental health, and the overall scores for male and female thalassemia patients are reported in Table 3. A statistically significant difference was only seen in Bodily Pain and Physical Health Summary, such that the male patients showed significantly higher scores than the female patients (*p-*value = 0.002, *p-*value = 0.046), respectively. There were no significant differences between male and female patients regarding their general health, physical functioning, role physical vitality, mental health, role emotional, social functioning, and mental health summary.

**Table 3:** Health-related Quality of life (QoL) of thalassemia patients, according to Short Form (SF-36) score.

Domains	Total SF-36 score (mean ± SD)	Men (N = 193) (mean ± SD)	Women (N = 163) (mean ± SD)	*p*-value
General health	48.19 ± 16.94	49.07 ± 17.04	47.15 ± 16.81	0.3
Physical functioning	72.47 ± 27.01	73.94 ± 26.26	70.74 ± 27.85	0.3
Role-physical	51.05 ± 37.72	53.24 ± 37.74	48.47 ± 37.75	0.2
Bodily pain	71.52 ± 27.56	75.63 ± 24.99	66.65 ± 29.67	0.002*
Vitality	59.44 ± 19.75	59.66 ± 19.76	59.17 ± 19.80	0.8
Mental health	63.47 ± 18.80	65.06 ± 18.36	61.60 ± 19.21	0.084
Role-emotional	58.52 ± 38.59	58.20 ± 39.28	58.89 ± 37.88	0.9
Social functioning	44.24 ± 23.33	44.02 ± 24.09	44.49 ± 22.47	0.9
Physical health	64.54 ± 18.04	66.29 ± 17.49	62.47 ± 18.52	0.046*
Mental health	57.92 ± 16.93	58.42 ± 17.06	57.32 ± 16.81	0.5
Total SF-36	61.24 ± 16.68	62.37 ± 16.58	59.90 ± 16.74	0.2

*p-value ˂ 0.05 was considered statistically significant.

### Covariates associated with SF-36 among patients in the univariate analysis

The analysis of variance (ANOVA) to determine the differences in physical health, mental health, and total SF-36 scores between groups are presented in Table 4. No statistically significant variations were found between scores, level of education, occupational status, economic status, and diagnosis (*p-*value > 0.05).

**Table 4:** Univariate analysis of covariates associated with Short Form-36 (SF-36) scores among thalassemia patients.

	Characteristics	Physical health	*p*-value	Mental health	*p*-value	Total SF-36 score	*p*-value
Level of education	Bachelor	61.93 ± 21.36	0.2	57.20 ± 17.55	0.6	59.57 ± 18.26	0.4
	Can read and write	57.30 ± 19.41		54.53 ± 18.65		55.91 ± 18.40	
	High school	67.06 ± 18.15		59.37 ± 16.48		63.22 ± 16.60	
	Illiterate	62.14 ± 12.06		51.27 ± 10.32		56.71 ± 10.41	
	Masters	64.00 ± 10.14		62.79 ± 11.13		63.40 ± 8.81	
	Primary	63.70 ± 16.64		57.72 ± 18.05		60.72 ± 16.48	
	Secondary	63.94 ± 18.18		56.12 ± 17.05		60.04 ± 16.97	
Occupational status	Civil servant	75.70 ± 0	0.15	59.20 ± 0	0.4	67.40 ± 0	0.2
	High school student	66.59 ± 17.79		59.65 ± 18.03		63.13 ± 17.21	
	Housewife	56.22 ± 14.69		51.46 ± 12.72		53.84 ± 12.35	
	Self-motivated	56.22 ± 14.69		58.71 ± 15.96		62.14 ± 15.84	
	Unemployed	63.25 ± 18.59		56.58 ± 16.44		59.92 ± 16.68	
	University students	59.29 ± 19.93		55.44 ± 14.59		57.39 ± 15.73	
Economic status	High income	44.37 ± 44.15	0.15	45.40 ± 43.28	0.2	44.90 ± 43.70	0.2
	Low income	64.83 ± 17.56		57.08 ± 17.36		60.96 ± 16.72	
	Moderate income	64.60 ± 17.94		59.08 ± 15.81		61.84 ± 15.98	
Diagnosis	Beta thalassemia	65.48 ± 17.42	0.2	59.09 ± 17.82	0.2	62.29 ± 16.86	0.15
	E-beta thalassemia	63.71 ± 18.29		57.00 ± 16.55		60.36 ± 16.58	
	Hemoglobin	75.23 ± 27.31		71.17 ± 20.15		73.20 ± 23.73	
	Others	76.57 ± 9.84		68.27 ± 9.14		72.43 ± 8.93	

### Factors associated with SF-36 among patients with thalassemia in the multivariate linear regression analysis

Multiple linear regression analysis was conducted to identify factors associated with HRQoL of thalassemia patients (Table 5). The socio-economic class (low and medium) was significantly negatively associated with the MH (*p-*value < 0.05). Age was negatively associated with GH, PF, RP, VT, RE, and MH but positively associated with BP. Male sex was positively associated with GH, PF, RP, BP, RE, and MH but negatively associated with VT. Participants diagnosed with E-beta thalassemia were negatively associated with GH, PF, and VT, but h-hemoglobin was positively associated with GH, PF, and VT (*p-*value > 0.05). Finally, the severity of the disease was positively associated with GH and BP but negatively associated with PF, RP, and VT (*p-*value > 0.05).

**Table 5:** Multiple linear regression analysis on factors of the Short Form (SF-36) scores among thalassemia patients.

Dependent variables	Independent variable	*p*-values	Beta	95% CI
GH	Age	0.007*	− 0.50	− 0.86, − 0.14
	Male	0.5	1.2	− 2.6, 5.0
	Diagnosis (E-β thalassemia)	> 0.9	− 0.26	− 4.3, 3.8
	Diagnosis (haemoglobin H disease)	0.2	11	− 8.0, 31
	Severity	< 0.001*	7.4	3.5,11
	Transfusion independent	0.046	8.7	0.16, 17
	Comorbidities (diabetes mellitus)	0.3	− 10	− 31, 9.9
	Comorbidities (hepatitis B or C)	0.4	6.2	− 8.1, 21
PF	Age	0.4	− 0.23	− 0.79, 0.33
	Male	0.3	3.4	− 2.5, 9.3
	Diagnosis(E-β thalassemia)	0.9	− 0.59	− 6.9, 5.7
	Diagnosis (haemoglobin H disease)	0.5	12	− 19, 42
	Transfusion independent	0.002*	22	8.2, 35
	Splenectomy (yes)	0.006*	− 8.9	− 15, − 2.6
	Severity	0.12	− 4.8	− 11, 1.3
RP	Age	0.11	− 0.66	− 1.5, 0.15
	Male	0.2	5.3	− 3.3, 14
	Occupation (unemployed)	0.7	− 15	− 102, 71
	Severity	0.3	− 4.6	− 13, 4.2
BP	Age	0.8	0.07	− 0.52, 0.65
	Male	0.020*	7.3	1.1, 13
	Severity	0.9	0.55	5.8, 6.9
	Comorbidities (diabetes mellitus)	0.032*	− 36	− 69, − 3.2
	Comorbidities (hepatitis B or C)	> 0.9	− 1.4	− 25, 22
	Transfusion independent	0.4	− 5.4	− 19, 8.6
VT	Age	0.4	− 0.18	− 0.61, 0.26
	Male	> 0.9	− 0.23	− 4.8, 4.4
	Diagnosis(E-β thalassemia)	0.6	− 1.4	− 6.3, 3.5
	Diagnosis (haemoglobin H disease)	0.4	9.2	− 14, 33
	Severity	0.7	− 1.0	− 5.7, 3.8
SF	Area of residence (urban)	0.4	− 2.4	− 7.6, 2.8
RE	Age	0.3	− 0.47	− 1.3, 0.37
	Male	0.8	0.86	− 8.0, 9.7
	Level of education (can read and write)	0.2	17	− 7.3, 41
	Level of education (high school)	0.9	− 1.6	− 22, 19
	Level of education (illiterate)	0.4	− 15	− 49, 20
	Level of education (master of science)	0.13	23	− 6.3, 51
	Level of education (primary school)	> 0.9	0.90	− 21, 23
	Level of education (secondary school)	0.7	− 4.7	− 26, 17
	Area of residence (urban)	0.4	3.4	− 5.0, 12
MH	Age	0.5	− 0.14	− 0.55, 0.27
	Male	0.2	2.8	− 1.5, 7.1
	Type of family (joint)	0.3	11	− 11, 33
	Type of family (nuclear)	0.14	17	− 5.2,38
	Area of residence (urban)	0.4	1.8	− 2.3,5.8
	Economic class (low income)	0.058/0.06	− 21	− 43, 0.74
	Economic class (medium income)	0.039/0.04*	− 23	− 44, − 1.1

*p-values < 0.05 was considered statistically significant.

GH and PF were positively associated with the blood transfusion (independent) but negatively related to the BP, which was not statistically significant. The presence of chronic illness/diseases/Comorbidities (Diabetes mellitus) was negatively associated with GH, BP, and Comorbidities (hepatitis B and C) were positively associated with GH but negatively with BP. Participants with a history of splenectomy showed a significantly negative association with PF (*p-*value < 0.05). The area of residence was negatively associated with SF but positively associated with RE and MH. The level of education (can read and write, Master of Science, primary school) was positively associated, and the level of education (secondary school, illiterate, high school) was positively associated with RE. The type of family was positively associated with MH.

### Risk factors for low health-related quality of life among thalassemia patients

Table 6 summarizes findings from the multiple logistic regression model associated with SF-36 scores. The patients were stratified into two groups according to PCS and MCS with a cutoff point of 50 and then explored the relationship between the PCS, MCS, and their determinant factors (Tables 6, 7).

**Table 6:** Logistic regression analysis of thalassemia patients with mental health component summary.

Characteristic	OR	95% CI	*p*-value
Age of participants	0.97	0.95, 1.00	0.035*
Gender			
Male	1.80	1.15, 2.85	0.011*
Marital status			
Single	1.65	0.90, 2.98	0.10
Type of family			
Joint	3.46	0.32, 75.6	0.32
Nuclear	5.40	0.51, 118	0.17
Level of education			
Can read and write	0.89	0.30, 2.61	0.83
High school	1.49	0.64, 3.38	0.34
Illiterate	1.53	0.27, 12.0	0.65
Master of science	5.50	0.85, 109	0.13
Primary school	1.51	0.60, 3.73	0.37
Secondary school	1.07	0.42, 2.70	0.89
Occupational status			
High school student	0.00		0.99
Housewife	0.00		0.99
Self-employed	0.00		0.99
Unemployed	0.00		0.99
University student	0.00		0.99
Area of residence			
Urban	1.22	0.78, 1.91	0.39
Socio-economic class			
Low income	1.07	0.05, 11.4	0.96
Moderate income	1.15	0.05, 12.2	0.91
Diagnosis			
E-beta-thalassaemia	0.86	0.52, 1.41	0.57
Hemoglobin H	0.84	0.08, 18.4	0.89
Others	2,412,057	0.00, NA	0.98
Severity			
Yes	0.96	0.61, 1.50	0.85
Transfusion status			
Transfusion independent	1.76	0.78, 4.53	0.20
Frequency of blood transfusion			
Low (6–12 times/year)	1.60	0.95, 2.70	0.076
None	2.76	0.85, 12.4	0.12
Occasional (1–5 times/year)	0.81	0.43, 1.52	0.50
Previous 3 months transfusion			
Yes	0.95	0.49, 1.76	0.87
Splenectomy status			
Yes	0.74	0.46, 1.20	0.22
Comorbidities status			
Diabetes mellitus	0.10	0.00, 1.21	0.10
Hepatitis B or C	0.72	0.09, 4.12	0.72
No comorbidity	1.35	0.18, 7.08	0.73
Others	0.73	0.09, 4.27	0.74
Iron chelating therapy status			
Poor compliance	1.28	0.48, 4.06	0.64
Medical expense	1.00	1.00, 1.00	0.20

*p-values < 0.05 was considered statistically significant.

**Table 7:** Logistic regression analysis of thalassemia patients with physical health component summary.

Characteristic	OR	95% CI	*p*-value
Age of participants	0.97	0.95, 1.00	**0.035**
Gender			
Male	1.80	1.15, 2.85	**0.011**
Marital status			
Single	1.65	0.90, 2.98	0.10
Type of family			
Joint	3.46	0.32, 75.6	0.32
Nuclear	5.40	0.51, 118	0.17
Level of education			
Can read and write	0.89	0.30, 2.61	0.83
High school	1.49	0.64, 3.38	0.34
Illiterate	1.53	0.27, 12.0	0.65
Master of science	5.50	0.85, 109	0.13
Primary school	1.51	0.60, 3.73	0.37
Secondary school	1.07	0.42, 2.70	0.89
Occupational status			
High school student	0.00		0.99
Housewife	0.00		0.99
Self-employed	0.00		0.99
Unemployed	0.00		0.99
University student	0.00		0.99
Area of residence			
Urban	1.22	0.78, 1.91	0.39
Socio-economic status			
Low income	1.07	0.05, 11.4	0.96
Moderate income	1.15	0.05, 12.2	0.91
Diagnosis			
E-β thalassemia	0.86	0.52, 1.41	0.57
Haemoglobin H disease	0.84	0.08, 18.4	0.89
Others	2,412,057	0.00, NA	0.98
Severity			
Yes	0.96	0.61, 1.50	0.85
Transfusion status			
Transfusion independent	1.76	0.78, 4.53	0.20
Frequency of blood transfusion			
Low (6–12 times/year)	1.60	0.95, 2.70	0.076
None	2.76	0.85, 12.4	0.12
Occasional (1–5 times/year)	0.81	0.43, 1.52	0.50
Previous 3 months transfusion			
Yes	0.95	0.49, 1.76	0.87
Splenectomy status			
Yes	0.74	0.46, 1.20	0.22
Comorbidities status			
Diabetes mellitus	0.10	0.00, 1.21	0.10
Hepatitis B or C	0.72	0.09, 4.12	0.72
No comorbidity	1.35	0.18, 7.08	0.73
Others	0.73	0.09, 4.27	0.74
Iron chelating therapy status			
Poor compliance	1.28	0.48, 4.06	0.64
Medical expense	1.00	1.00, 1.00	0.20

Significant values are in bold.

Logistic regression analysis demonstrated that age (OR 0.97, CI 0.95–1.00, *p-*value = 0.035) and male gender (OR 1.80; CI 1.15–2.85; *p-*value = 0.011) were significant predictors of HRQoL (i.e., total summary score). However, there were no statistically significant relationships between the scores and other factors like marital status, type of family, occupation, education, splenectomy, severity, transfusion status, etc. (*p-*value > 0.05).

## Discussion

The significant findings of this study are the HRQoL of male patients is more vulnerable compared to that of females, and the overall HRQoL of TP is associated with lower income, high blood transfusion status, disease severity, comorbidities, and medical expenses. The majority of TP were transfusion dependent (91%), having comorbidities (26%), and from low-income families (52%).

Bangladesh is a low-middle-income country (World Bank: *Data for Bangladesh, Lower Middle Income|Data*, n.d.). Low income, high medical expenses, and high blood transfusion frequency significantly affect the HRQoL^29–31^. Evidence from previous studies reported that household income negatively impacted the HRQoL summary score^32^. Thalassemia patients with higher incomes scored higher in physical health, mental health, and total quality of life scores than those with lower incomes^33^. Low income was also associated with lower scores for physical health, mental health outcomes, and total SF-36 scores^33^. Low income and high medical costs can affect the HRQoL of thalassemia patients in several ways. Firstly, low income can lead to reduced access to healthcare facilities, limited disease management resources, and physical and mental health outcomes. Financial barriers prevent them from seeking necessary treatment in time, resulting in physical and psychological health outcomes and a deteriorating quality of life.

Furthermore, thalassemic patients require lifetime treatment, which requires high blood transfusions, iron chelation therapies, laboratory testing, and associated expenses affecting life satisfaction and quality of life^1,34–36^. In addition, increased medical costs can cause significant financial strain leading to stress, anxiety, and poor quality of life for thalassemia patients and their families^37^. High blood transfusion frequency significantly affects the HRQoL. However, there was no significant association found with transfusion frequency reported^38^. High blood transfusion frequency is another factor that can impact the HRQoL of thalassemia patients. While regular blood transfusions are essential for maintaining health in thalassemia patients, they can also lead to complications such as iron overload, negatively impacting organ function and physical health outcomes.

Furthermore, frequent transfusions can be time-consuming and require patients to take time off work or school, affecting their daily routine and social interactions and reducing HRQoL scores^30,39^. Clinicians and policymakers should be aware of these factors and address them to improve HRQoL outcomes for thalassemia patients. In addition, 26% of the patients had comorbidities, including cardiological disorders, diabetes mellitus, hepatitis B or C, and other related complications. The presence of comorbidities was an independent factor for lower physical health summary scores and overall quality of life of the patients^7,40^. Reportedly, thalassemic patients with comorbid medical conditions demonstrated substantially impaired reduced HRQoL^41^. Comorbidities attributed to the increased burden of managing multiple health conditions, including frequent medical visits, increased healthcare costs, and the potential adverse effects of medicines^42^. Moreover, comorbidities may lead to increased complications and worsening of symptoms of thalassemia, further reducing the physical health and QoL of patients^43^.

The study showed that most participants (68%) suffered from Hb E/β-thalassemia. More than half (54%) of the participants had a severe form of the disease, and approximately 91% were transfusion dependent. All domains of HRQoL were compromised in patients with thalassemia. In addition, poor iron chelating therapy compliance was also associated with poor physical health, mental health, and total SF-36 scores. Our findings showed that age was negatively correlated with the quality of life among patients with thalassemia, which was statistically significant. Previous studies have stated a similar observation^19,38,44–46^. Due to disease-related complications and social psychological challenges, age is negatively correlated with quality of life (QoL) in thalassemia patients. Elderly thalassemia patients may experience reduced mobility, social isolation, anxiety related to disease progression, and decreased resilience and coping mechanisms. Older thalassemia patients had lower QoL scores than younger patients, with disease-related comorbidities and complications potentially explaining this finding.

There was no significant difference in mental and physical health between males and females. However, men scored higher than women on one scale for bodily pain (BP) (*p* = 0.002). A previous study in South-West Iran^46^ observed no significant association between gender and overall quality of life. However, our observations were echoed by a few earlier studies^44,45,47^, reporting that gender was correlated with overall HRQoL and mental and physical health scores. One possible explanation for gender-related differences in HRQoL and health outcomes among thalassemia patients could be related to differences in disease severity. Research has shown that males with thalassemia tend to have more severe symptoms and require more frequent transfusions than females. This could lead to a more significant impact on their physical health and quality of life^48,49^.

No statistically significant association was observed between other indices (Family type, marital status, education level, and economic class) and HRQoL. These findings were consistent with a previous study^46^ suggesting married persons scored higher in mental and physical health. There are several possible explanations for why marital status may be associated with higher health-related quality of life (HRQoL) scores in thalassemia patients. Firstly, marriage may offer emotional support and a sense of belonging, positively affecting mental health and overall well-being. Secondly, spouses may provide practical assistance with treatment adherence and management of the illness, which can lead to better physical health outcomes. Lastly, married individuals may have greater access to social and financial resources, which can also positively impact HRQoL. These factors suggest that marital status is an essential consideration for healthcare providers when managing the well-being of thalassemia patients. Ensuring unmarried patients receive appropriate support and resources to address the challenges of living with the illness is essential. However, education level was significantly associated with many aspects of HRQoL in earlier research^45^. However, insignificant income was correlated with physical health, mental health, and Quality of life scores^46^. The impact of income level on the health-related quality of life (HRQOL) of thalassemia patients may be attributed to several factors. Firstly, the cost of thalassemia treatment, which involves frequent medical interventions, including blood transfusions and iron chelation therapy, may be financially demanding for low-income patients. Consequently, the financial burden may lead to poor adherence to treatment, resulting in the exacerbation of symptoms and deterioration of both physical and mental health. Secondly, patients with low income may experience social and emotional stress, which may adversely affect their mental health and overall quality of life.

The logistic regression analysis demonstrated that comorbidities were not significant predictors of HRQoL. However, this finding was inconsistent with previous studies indicating that a comorbid condition in thalassemic patients is significantly associated with the overall quality of life^44,45,47^.

The clinical characteristics (clinical subtype, disease severity, splenectomy status, blood transfusion, and iron-chelating therapy) showed no significant association with mental and physical health components. Nonetheless, evidence showed that patients having a severe disease condition, a history of splenectomy, and receiving a frequent transfusion and iron-chelating medications had significantly poor HRQOL scores^45,50–52^. The contribution of factors such as disease severity, history of splenectomy, regular blood transfusions, and iron chelation therapy to poor health-related quality of life (HRQoL) in thalassemia patients can be attributed to several reasons. Firstly, a severe disease condition may cause physical and emotional stress, leading to a decline in HRQoL. Secondly, a history of splenectomy may increase the likelihood of developing infections and other complications, further impacting HRQoL. Thirdly, frequent blood transfusions and iron chelation therapy may be time-consuming and costly, resulting in side effects such as pain, discomfort, and fatigue, which can negatively impact HRQoL.

Thalassemia can be fatal if the patient is not appropriately treated^53–56^. However, the outlook of thalassemia has remarkably transformed in the recent few years from a life-threatening deadly disease to a chronic illness with disability^57^. Clinical management has improved extensively in recent years, even in developing countries; however, minimal attempts are made to uplift the quality of life among patients^58^. Thus, assessing the HRQoL in patients with thalassemia is crucial, as identifying factors that lead to poor quality of life.

In addition to physical and mental health issues, out-pocket expenditure for treating thalassemia can cause financial and social problems. These may lead to poor quality of life or poor chances of leading happy lives. Thus, these patients require physical rehabilitation and a psychosocial support network that can give them hope for a better future. Policymakers in this field should pay special attention to patients with comorbid conditions, poor compliance with iron chelating therapy, and lower socio-economic status to improve their 'Quality of life (QoL). In addition, family members should support them in overcoming mental health issues besides physical illness. However, further nationwide research is needed to depict the real scenario regarding the quality of life of thalassemia patients. The present study is a single-center cross-sectional study; thus, this was not nationally representative data.

## Conclusions

The findings of this study highlighted the significant negative impact of thalassemia and its treatment on HRQoL in terms of comorbidities, poor iron chelating therapy, and poor income. This study suggested that modifying existing thalassemia management and care could be beneficial. Psychosocial and counseling programs aimed at helping patients discuss and accept their illness, facilitating a normal lifestyle, and providing a link between patients, school officials, the family, and the physician might help alleviate these difficulties, especially academic performance. In addition, modification of health care services for children with thalassemia to become more patient-centered, flexible, and comprehensive reduces time spent at hospitals. It improves treatment outcomes, including the HRQoL of the patients. In the future, several potential therapies could significantly impact treating thalassemia patients. Gene therapy and stem cell therapy are promising therapies for developing the treatment of thalassemia. These therapies have the potential to offer long-term cures, reduce the need for blood transfusions, and improve the quality of life for patients with thalassemia. Patients must stay informed and work with their healthcare providers to explore all available treatment options available^59–62^.

## Supplementary Information

Supplementary Information.

## Data Availability

The data used in this study will be available upon request to the corresponding author.
